# Toward European harmonization of national myasthenia gravis registries: modified Delphi procedure-based expert consensus on collectable data

**DOI:** 10.1186/s13023-024-03520-3

**Published:** 2025-03-11

**Authors:** Abderhmane Slioui, Giulia Tammam, Fiammetta Vanoli, Adela Della Marina, Stanislav Vohanka, Nils Erik Gilhus, Isabella Moroni, Maria Isabel Leite, Fredrik Piehl, Carlo Antozzi, Jonathan Pini, Frauke Stascheit, Shahram Attarian, Ernestina Santos, Jan Verschuuren, Lou Canonge, Jeremy Garcia, Caroline Perriard, Elena Cortés-Vicente, Renato Mantegazza, Andreas Meisel, Sabrina Sacconi

**Affiliations:** 1 Peripheral Nervous System and Muscle Department, Reference Center for Neuromuscular Disorders, Pasteur 2 Hospital, Centre Hospitalier, Universitaire de Nice, Nice University Hospital, SNPM – Hôpital Pasteur 2 - 30 voie Romaine, 06001 Nice CEDEX, France; 2 Department of Brain and Behavioral Sciences, University of Pavia, IRCCS Mondino Foundation, Pavia, Italy; 3 Neuroimmunology and Neuromuscular Diseases Unit, Fondazione IRCCS Istituto Neurologico Carlo Besta, Milan, Italy; 4 Department of Human Neurosciences, Sapienza University of Rome, Rome, Italy; 5 Department of Pediatric Neurology, Centre for Neuromuscular Disorders, C-TNBS, University Duisburg-Essen, Essen, Germany; 6 Department of Neurology, Faculty of Medicine, University Hospital Brno, Masaryk University, Brno, Czechia; 7 Department of Clinical Medicine, University of Bergen, Bergen, Norway; 8 Department of Pediatric Neurosciences, Fondazione IRCCS Istituto Neurologico Carlo Besta, Milan, Italy; 9 Nuffield Department of Clinical Neurosciences, University of Oxford, John Radcliffe Hospital, Level 3, West Wing, Headley Way, Oxford, OX3 9DU UK; 10 Departments of Clinical Neuroscience, Karolinska Institutet, and Neurology, Karolinska University Hospital, Stockholm, Sweden; 11 Immunotherapy and Apheresis Departmental Unit, Fondazione IRCCS Istituto Neurologico Carlo Besta, Milan, Italy; 12 Corporate Member of Freie Universität Berlin and Humboldt Universität Zu Berlin, Department of Neurology With Experimental Neurologie, Neuroscience Clinical Research Center, Universitätsmedizin Berlin, Charitéplatz 1, 10117 Berlin, Germany; 13 Reference Center for Neuromuscular Disorders and ALS, Timone University Hospital, Aix-Marseille University, Marseille, France; 14 Neurology Department, Centro Hospitalar Universitário de Santo António; Unit for Multidisciplinary Research in Biomedicine, Instituto de Ciências Biomédicas Abel Salazar, Universidade do Porto, Porto, Portugal; 15 Department of Neurology, Leiden University Medical Center, Leiden, the Netherlands; 16 ESIEE PARIS School, Gustave Eiffel University, Paris, France; 17 Reference Center for Neuromuscular Disorders, Lenval Pediatric Hospitals of Nice University Hospital, Nice, France; 18 Neuromuscular Diseases Unit, Hospital de La Santa Creu I Sant Pau, Barcelona, Spain; 19 Institute for Research On Cancer and Aging of Nice, CNRS, INSERM, Côte d’Azur University, SNPM – Hôpital Pasteur 2 - 30 voie Romaine, 06001 Nice CEDEX, France

**Keywords:** Rare disease registry, Delphi procedure, Myasthenia gravis, European registry, Expert panel

## Abstract

**Background:**

Myasthenia gravis (MG) is a rare autoimmune disorder. Several new treatment concepts have emerged in recent years, but access to these treatments varies due to differing national reimbursement regulations, leading to disparities across Europe. This highlights the need for high-quality data collection by stakeholders to establish MG registries. A European MG registry could help bridge the treatment access gap across different countries, offering critical data to support regulatory decisions, foster international collaborations, and enhance clinical and epidemiological research. Several national MG registries already exist or are in development. To avoid duplication and ensure harmonization in data collection, a modified Delphi procedure was implemented to identify essential data elements for inclusion in national registries.

**Results:**

Following a literature review, consultations with patient associations and pharmaceutical companies, and input from multiple European MG experts, 100 data elements were identified. Of these, 62 reached consensus for inclusion and classification, while only 1 item was agreed for exclusion. 30 items failed to reach the ≥ 80% agreement threshold and were excluded. Among the 62 accepted items, 21 were classified as mandatory data elements, 32 optional, and 9 items pertained to the informed consent form.

**Conclusions:**

Through a modified Delphi procedure, consensus was successfully achieved. This consensus-based approach represents a crucial step toward harmonizing MG registries across Europe. The resulting dataset will facilitate the sharing of knowledge and enhance European collaborations. Furthermore, the harmonized data may assist in regulatory or reimbursement decisions regarding novel therapies, as well as address treatment access disparities between European countries.

## Background

Myasthenia Gravis (MG) is a rare, heterogeneous neuromuscular junction (NMJ) disorder, characterized by muscle weakness and fatigability. It is an autoimmune disease driven by antibodies targeting components of the post-synaptic endplate at the NMJ [1, 2]. The majority of antibodies are directed against the acetylcholine receptor (AChR) [1–4], while less commonly they target muscle-specific tyrosine kinase (MuSK) [5–7] or lipoprotein related protein 4 (LRP4) [8–10]. MG is classified into subtypes based on clinical presentation, age of onset, autoantibody profile, and thymic involvement, all of which influence therapeutic responses to specific treatments [3, 10–17]. Symptom localization further divides MG into ocular or generalized forms. The variability in symptom severity and distribution from patient to patient contributes to the disease’s heterogeneous clinical presentation [18].

The incidence of MG varies across studies, ranging from 1.7 to 21.3 cases per million people and year, with a reported prevalence between 15 and 234 per million [19]. MG affects both sexes, though there is a predominance of early-onset cases in females and late-onset cases in males [20, 21]. As populations age, the prevalence of late-onset and very-late-onset MG is increasing, reshaping the disease’s epidemiology and presenting unique management challenges for these subpopulations, who are at greater risk of iatrogenic complications and comorbidities [22].

Approximately 80% of MG patients present with anti-AChR antibodies, while the remaining 20% include those with anti-MuSK or anti-LRP4 antibodies, or patients without detectable antibodies by routine testing, known as seronegative MG [3, 23]. Other antibodies, such as anti-titin, anti-ryanodine receptor, or anti-agrin, can be detected in a minority of patients, typically in association with thymomas [24–31].

Pyridostigmine remains the first-line symptomatic treatment for MG. Immunosuppressive therapy is frequently initiated in conjunction, with prednisone being the preferred first-line agent due to its rapid effects, often accompanied or followed by a steroid-sparing immunosuppressive drug [32]. During myasthenic crisis, intravenous immunoglobulins and plasma exchange are used [33]. Many patients continue to experience MG-related symptoms or adverse iatrogenic effects, leading to diminished quality of life and adding to a high burden of disease [34].

Over the past decade, new therapeutic options have emerged, providing second- and third-line treatment alternatives for patients who either do not respond to or cannot tolerate conventional therapies leading to ongoing, not controlled disease. However, access to these novel treatments is frequently restricted due to approval indications, their high costs and variations in national reimbursement policies, resulting in significant disparities in access to care across Europe. A growing number of national MG registries have been established across Europe, with financial support from health authorities’, pharmaceutical companies and patient associations with the aim of collecting data on the long-term effects of these new treatments and to better characterize and understand the complexity and the diversity of the disease.

However, despite the creation of several national MG registries, differences between countries still exist, particularly in terms of access to novel therapies. In order to address disparities in the diagnosis and treatment of MG, it is fundamental to highlight the differences between countries, and this inevitably requires a harmonization of registries across countries.

Given a fragmented landscape at the national level, the establishment of a European MG registry presents a unique opportunity to address disparities in the diagnosis and treatment of MG across different countries, particularly in terms of access to novel therapies. It would also help to assess differences in disease epidemiology, demographics, disease expression, and standards of care in different European countries. A European registry would not only help to overcome inequalities in treatment access but would also provide critical data on the safety and efficacy of emerging therapies, which can be instrumental in regulatory decision-making and would ensure a comparable management of MG patients in all European countries. Additionally, such a registry would foster international collaborations, thereby advancing clinical, pharmaceutical, and epidemiological research as well as quality control.

To align the vision of a European registry with the current reality of national registries, data harmonization across these registries is essential. The standardization of data collection among different countries represents a key step towards the creation of a European registry. In this study, we aimed to identify a common dataset to be used in the national autoimmune MG registries in order to achieve harmonization between European countries. A multidisciplinary approach, comprehensive of collaboration with different European MG experts, patient associations and pharmaceutical companies, was used to identify potential data elements for inclusion in the registry, and then a modified Delphi method was used to reach consensus. Here we present the results of this multidisciplinary consensus-based approach.

## Methods

**Fig. 1:**
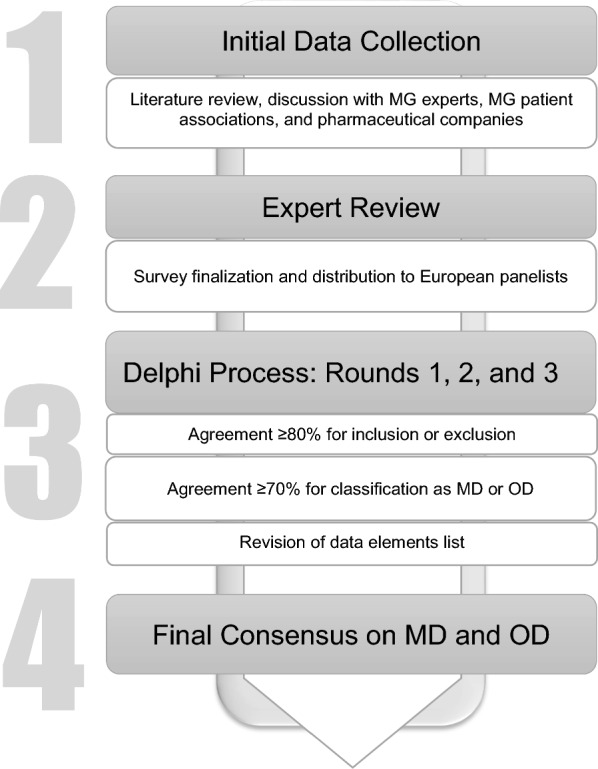
Schematic representation of the modified Delphi procedure employed: representation of the modified Delphi procedure used for the selection of items of the National Registry of Myasthenia Gravis Patients: collection of potential items through 4 different approaches; revision of data and finalization of survey; 3 consecutive Delphi rounds to gather the expert panel's opinions and achieve consensus on the selected items (MD: Mandatory Data, OD: Optional Data)

The Delphi method is an iterative, multistage process aimed at obtaining the consensus opinion of a group of experts on a specific topic [35, 36], and is widely recognized as an effective tool for solving complex problems in healthcare. Traditionally, the Delphi procedure involves multiple rounds of questionnaires aimed at achieving consensus on selected topics. Our approach began with an extensive literature and database search, followed by consultations with patient associations and pharmaceutical companies, as well as expert group discussions to identify key topics and relevant items related to autoimmune MG from national MG registries for inclusion in the Delphi procedure. Notably, congenital myasthenic gravis and Lambert-Eaton myasthenic syndrome were excluded in this Delphi procedure. A schematic representation of the modified Delphi procedure we employed is provided in Fig. 1.

### Expert panel selection

The expert panel comprised leading specialists in MG who were involved in national MG registries across Europe. Special attention was given to ensuring broad geographical representation of the European registry initiatives. The panel included 11 neurologists, 4 pediatric neurologists, and 1 physiotherapist from 11 European countries (Belgium, Czech Republic, France, Germany, Italy, Netherlands, Norway, Portugal, Spain, Sweden, and United Kingdom). Most of them are members of the European Reference Network for Neuromuscular Diseases (ERN-NMD). These experts also participated in the 278th ENMC International Workshop, titled "European standards for harmonization of myasthenia gravis (MG) registries and emerging digital solutions." Two neurologists (SS and FV) served as facilitators for the panel.

### Literature and database review

We conducted a comprehensive search of studies published between January 2010 and December 2023 in PubMed, the Cochrane Library, and EMBASE. The MeSH search terms included "myasthenia gravis registry," "outcome measures," and "myasthenia gravis therapeutic trials." English-language articles were screened for eligibility based on title, abstract, and full-text availability. The inclusion criteria were evaluated by three authors (LC, AS, and SS).

### Patient associations and pharmaceutical companies

Representatives from 6 patient advocacy organizations, including AFM Telethon (Myasthenia Group), Asociación Miastenia de España (AMES), Belgian Association against Neuro-Muscular Diseases (Myasthenia Group), Deutsche Myasthenie Gesellschaft, EURORDIS, and the Italian Association of Myasthenia Gravis, were engaged in group discussion preceding the Delphi panel. The main objective was to identify patient priorities and incorporate their perspectives on the harmonization of national MG registries. A questionnaire with four open-ended questions was then distributed to the representatives to assess their opinions on which data elements should be included in the registry.

A similar group discussion was held with representatives from four pharmaceutical companies active in MG research and development (Alexion Pharmaceuticals, argenx, Johnson & Johnson, and UCB Pharma). These representatives were also sent a questionnaire with four open-ended questions to gather their input on the data elements for the MG registry. The responses from all stakeholders were qualitatively analyzed to ensure that the proposed Delphi items addressed their concerns and requirements.

### Delphi design and survey

Based on the findings from the literature review, patient association input, and pharmaceutical consultations, a preliminary questionnaire was developed by AS, GT, and SS. This draft was then reviewed by three experts (AM, ECV, and RM), who provided feedback. The finalized survey was subsequently distributed to a European panel of MG experts and subjected to three rounds of the Delphi process.

Online surveys were created using Google Forms and distributed via email to the experts. Responses were anonymized for each round. The survey consisted of 100 questions related to the inclusion of specific data elements, and the experts were asked to rate their agreement on a scale of 1 (complete disagreement) to 5 (complete agreement). For items that achieved consensus, the experts were also asked to define the item's relevance. Items were classified as either Mandatory (MD) or Optional (OD). MD elements were defined as essential for uniformly collecting patient data, particularly for understanding the individual MG history and guiding treatment development. OD elements were considered additional non-essential data. The primary distinction between OD and MD was the time and resources required for data collection. To ensure comprehensive patient documentation, it was necessary to limit the number of mandatory information items. All data elements, except those related to the informed consent form (ICF), were categorized as either MD or OD.

**Fig. 2:**
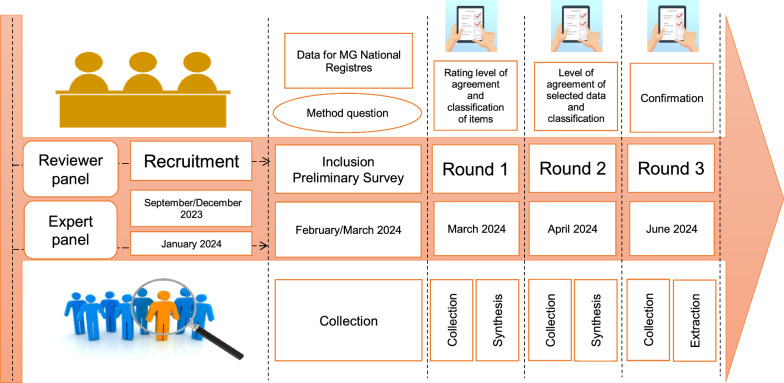
Timeline of meetings and surveys of the modified Delphi procedure: schematic representation of all meetings and surveys organized for the modified Delphi procedure

Three consecutive Delphi rounds were conducted to gather the expert panel's opinions on the data elements. Consensus was defined as ≥ 80% agreement among panelists for the inclusion or exclusion of an item. For an item to be classified as MD or OD, a consensus of ≥ 70% was required. Items that failed to achieve consensus were reintroduced in subsequent rounds. The rounds were concluded when the results showed no significant differences from the previous round. A final discussion was held among the panelists and facilitators to finalize the consensus. All meetings and surveys of the Delphi procedure were organized according to a pre-established agenda (Fig. 2).

## Results

Overall, 100 unique items were identified through a comprehensive literature review, discussions with patient associations and pharmaceutical companies, and expert consultations. Of these, 9 items pertained to the ICF.

### Literature review

A total of 623 studies were identified through the literature search. After removing duplicates and reviewing titles and abstracts, 348 studies were selected for further evaluation. Following a full-text analysis, SS, LC, and AS selected 54 studies. These included 7 retrospective studies, 9 prospective studies, 4 qualitative studies, 5 validation studies, 27 literature reviews, and 2 commentaries. Each of these studies was used to identify relevant items for inclusion in the registry. The items derived from these 54 studies covered key areas such as patient demographics, laboratory tests, diagnostic procedures, and clinical data. These items were subsequently classified and grouped to facilitate further analysis, resulting in the identification of 73 potential items for the MG registry. A detailed list of the selected articles is provided in the supplementary material (Supplementary Table 1).

### Assessment of the perspectives of patient associations and pharmaceutical companies

Patient representatives emphasized the need for obtaining prior consent for data collection and utilization, noting the variations in legal requirements across different countries. They stressed the importance of transparency and providing patients with comprehensive information prior to enrollment in national registries, suggesting that this process should be managed by registry coordinators or physicians. Furthermore, patient representatives advocated for the formation of a dedicated scientific committee to evaluate future projects involving the data registry, and they highlighted the importance of involving patients in decision-making processes regarding the registry’s development on national and European levels.

Additionally, patient representatives proposed the inclusion of several data elements, such as social interactions, comorbidities, physical activity, family history of MG, and professional activities, as these factors were considered essential for a holistic understanding of patient experiences and outcomes.

Pharmaceutical companies emphasized the need to include data elements that would provide evidence of treatment effectiveness and safety in *real-world* clinical settings. They also recommended items that would allow for comparisons across different countries and practices. A particular focus was placed on underrepresented populations, such as older, frail, and comorbid patients often excluded from clinical trials. The inclusion of data on these populations was seen as critical for improving treatment understanding and outcomes.

As a result of these discussions, 14 additional items were proposed. Expert discussions subsequently yielded 13 additional items and helped refine some of the previously proposed items to avoid redundancy.

### Delphi rounds

All panel members participated in the first two rounds of the Delphi process, and all but one participated in the third round.

In the first Delphi round, consensus was achieved for 62 out of 100 items, with an agreement level of ≥ 80%. Among these, only 18 items reached ≥ 70% agreement regarding their classification as MD or OD. Specifically, 10 items were designated as MD, and 8 as OD. One item was deemed unnecessary for inclusion in the registry, while all 9 ICF-related items received ≥ 80% agreement for inclusion (Table 1).

**Table 1:** Items of the informed consent form (ICF)

Items
1. The Informed Consent Form (ICF) for participants in MG national registries must be accepted by all European Ethics Committees and address Ethical, Legal and Social Implications (ELSI) issues (specifically GDPR compliance) while providing a harmonized framework for European registry exploitation approval
2. A pediatric version of the ICF (including age-specific Information Sheets and Assent Forms) must be available
3. The ICF must outline data use conditions, including those for research purposes at the national level
4. The ICF must outline data use conditions, including those for research purposes at European level
5. The ICF must outline data use conditions, including those for data transfer outside Europe
6. The ICF must outline data use conditions for commercial purposes
7. The ICF must specify how patients will be informed about future changes in data collection
8. The ICF must specify how patients will be contacted to participate in research projects
9. The ICF must include information on data ownership

List of the 9 items pertained to the ICF

The 38 items that did not achieve consensus in the first round were reintroduced in the second Delphi round. Additionally, the 34 items without consensus on classification at the first round were re-evaluated. By the end of the second round, an additional 5 items reached consensus for inclusion, increasing the classified items to 21 for MD and 18 for OD.

In the third and final Delphi round, 3 more items achieved ≥ 80% agreement for inclusion in the registry, while 14 additional items were classified as OD. A total of 30 items failed to achieve ≥ 80% consensus and were excluded from the registry. 7 items received ≥ 80% agreement for inclusion but did not meet the required ≥ 70% consensus for either MD or OD, thus remaining unclassified after discussions with the panelists.

**Fig. 3:**
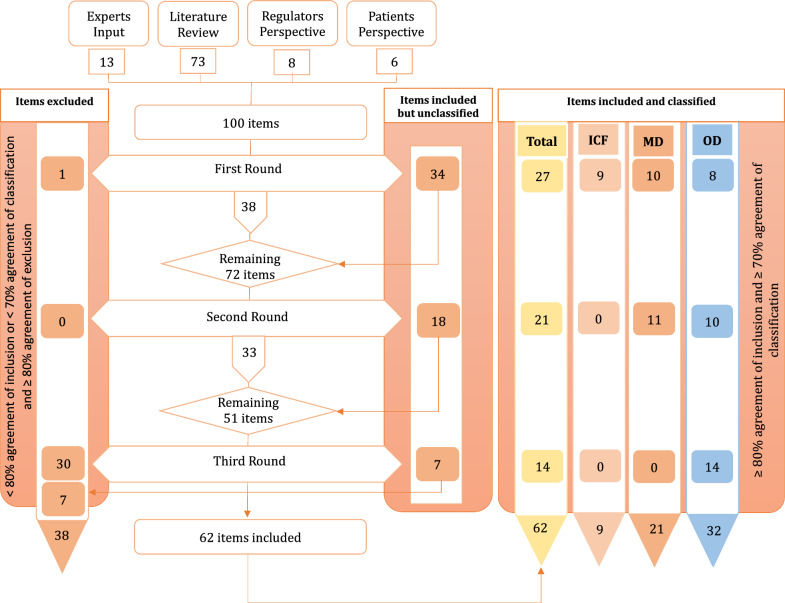
Representation of the Delphi round results: detailed representation of the results of each Delphi rounds, classified into optional and mandatory data elements

At the end of the Delphi process, consensus was achieved for the inclusion of 62 items in the national registry. Of these, 21 were classified as MD, 32 as OD, and 9 pertained to the ICF. However, 7 items remained unclassified due to divergent opinions among the panelists and were subsequently excluded. A detailed schematic representation of Delphi round results is provided in Fig. 3. A complete list of all classified MD and OD items is presented in Table 2, while Table 3 lists the excluded and unclassified items.

**Table 2:** Items classified as mandatory and optional data elements at the final Delphi round

Mandatory data (MD)	Optional data (OD)
Reference center information	Referring clinician information
Sex of the patient	Country of origin of the patient
Gender of the patient	Current employment/schooling
Date of birth of the patient	Degree of disability
Date of MG onset	Method of antibody detection
Type of first symptom	MGFA at the enrollment in the registry
Date of diagnosis	Year of thymus exploration
MG diagnosis (ocular or generalized form)	Thymectomy date
Antibody type (Anti-Rach, Anti-Musk, Anti-LRP4, seronegative)	Thymectomy technique
Repetitive nerve stimulation (RNS) results	WHO classification of thymoma
MGFA at the diagnosis (MGFA-D)	MASAOKA-KOGA staging of thymoma
Thymus explored (Yes/No)	Comorbidities: autoimmune diseases
Results of thymus exploration (Normal, thymus hypoplasia, thymoma)	Comorbidities: metabolic diseases
Thymectomy	Comorbidities: cardiovascular diseases
Histology of thymus	Comorbidities: lung diseases
Consultation date	Comorbidities: neurological diseases
MG-ADL Score	Comorbidities: malignancies (other than thymoma)
Dose of MG related therapy(ies) and recurrence	Comorbidities: musculoskeletal diseases (other than MG)
MG related death	Comorbidities: Relation with MG (Yes/No)
Cause of death	Quantitative Myasthenia Gravis Scoring System (QMG)
CTIS (clinical trial number)	Myasthenia Gravis Composite Score (MGC Score)
	For each comorbidity, specify if related to MG therapy
	Family history of MG
	Patient body weight and height
	MGFA Clinical current status (MGFA-CS)
	Myasthenia Gravis Quality Of Life Score (MG-QOL15r)
	Class, name, and dosage of MG related therapy(ies)
	Hospitalisation for chronic MG related therapies
	Hospitalisation for MG crisis/ deteriorations
	Type of hospital unit (i.e. Emergency/ Neurology/ICU/Other)
	Hospitalisation for MG therapy side effects
	Home infusion for MG related therapy(ies)

Detailed list of the complete items classified as MD and OD elements at the end of the Delphi rounds

**Table 3:** Items excluded from the registry

List of items excluded from the registry	List of items not classified and excluded at the final Delphi round
Clinician experience in myasthenia gravis	SFEMG (Single Fiber Electromyography) results
Ethnic origin of the patient	Technique used for thymus exploration
City of residence	Type of consultation (Inpatient or outpatient)
Current marital status	If MG related therapy was stopped, specify the reason
Current level of education	Suspected cause of MG crisis /deterioration
Social care	Death date
Quantified antibodies results	Participation in clinical trials status
Pharmacological test anticholinesterase	
Type of pharmacological testing	
Psychiatric diseases	
Addiction disorders	
Gastrointestinal diseases	
Ophthalmological diseases	
Genito-urinary diseases	
ENT diseases	
Dermatological diseases	
Hematological diseases	
Surgical procedures (other than thymectomy)	
Family history of autoimmune diseases	
SF-36	
Fatigue Severity Score	
PROMIS Fatigue Score	
Hospital anxiety and depression score (HADS Score)	
Insomnia severity index	
Visual analogue pain intensity scale	
Localisation of pain	
List of all ongoing non-MG related pharmacological therapies and recurrence (date of onset,status,dosage,recurrence)	
List of ongoing non-pharmacological therapies type i.e. Physiotherapy, occupational therapy, psychological support, nutritional support, orthophonist, NIV…(date at onset,statut,recurrence..)	
If ongoing recurrence	
If no, wish to participate in clinical trial	

List of the items excluded from the registry at the end of the Delphi rounds (agreement < 80% for inclusion or agreement < 70% for classification)

### Informed consent form (ICF)

All 9 items related to the ICF received ≥ 80% agreement in the first Delphi round. These items are consistent with the European Reference Network (ERN) registry consent form for rare neuromuscular diseases [37]. A complete list of ICF items is provided in Table 1.

### Demographics and center information

Key patient demographic data, such as sex, gender, date of birth, the center where the patient is followed, and the date of the last visit, were classified as MD. Other items, including height, weight, country of origin, and current employment or educational status, were classified as OD. Information pertaining to the referring clinician was also classified as OD. To ensure compliance with general data protection regulation (GDPR), all panelists agreed that any data allowing for direct patient identification should be excluded. They emphasized the importance of encrypting any identifiers that connect individuals to stored data.

### Medical history

Certain clinical, laboratory, and electromyographic data, such as the date of disease onset and diagnosis, the characteristics of the first symptoms, Myasthenia Gravis Foundation of America (MGFA) classification at diagnosis, antibody presence and type, and repetitive nerve stimulation results, were classified as MD. Information regarding the cause of death and its relation to MG, if applicable, was also considered MD. Additional data, such as laboratory methods for antibody detection, hospitalizations due to MG crises or relapses, and severe side effects from MG therapies, were classified as OD. The panelists noted that these data might not always be collected and suggested that pharmacovigilance might be a more suitable mechanism for monitoring therapy-related safety.

### Thymus disease

Given the thymus’s critical role in the pathophysiology of early-onset MG with AChR antibodies and thymomas, data related to thymus disease were classified as MD. This classification includes thymectomy history and histological data. Additional information, such as the date of surgical intervention, thymectomy technique, WHO classification for thymoma, and staging according to the Masaoka-Koga scale, was classified as OD.

### MG scales to measure severity and quality of life

Accurate measurement of MG severity and its impact on patient’s daily activities and quality of life is crucial for clinical monitoring and treatment evaluation. Among the various scales evaluated, the Myasthenia Gravis Activities of Daily Living (MG-ADL) score was the only one measure classified as MD. This 8-item patient-reported outcome measure is widely recognized for its validity, reliability, and responsiveness [38] and has been used as a primary outcome measure in several clinical trials. The MGFA classification at diagnosis (MGFA-D) was also classified as MD. In contrast, other scales, including the MGFA Clinical Status (MGFA-CS), the Quantitative Myasthenia Gravis (QMG) score, the Myasthenia Gravis Composite (MGC) score, and the Myasthenia Gravis Quality of Life score (MG-QoL15r), were classified as OD. These scales are often time-consuming, reported heterogeneously, and not routinely used.

### MG therapies

As new treatments for MG become increasingly available, questions remain regarding their optimal use, indications, dosages, and risk–benefit profiles. Several therapy-related items were classified as MD, including dosage and recurrence of the MG therapy, while the class and/or name of the therapy was classified as OD. These data are critical for assessing and comparing the efficacy and safety of both novel and standard treatments. Participation in clinical trials and trial numbers were also classified as MD whereas data on home treatments were classified as OD.

### Comorbidities

Comorbidities, particularly in late-onset MG patients, can significantly affect disease progression and influence treatment decisions. The panel quickly agreed that most proposed comorbidities, including autoimmune, pulmonary, cardiovascular, metabolic, neurological, oncological, and musculoskeletal conditions, should be included in national registries. However, they were classified as OD. The panel also recommended recording whether comorbidities were related to MG therapies and whether they resulted in hospitalization.

### Excluded items

30 items did not achieve ≥ 80% consensus for inclusion in the national MG registry. Many of the excluded items related to comorbidities, such as psychiatric, gastrointestinal, ophthalmological, genitourinary, otorhinolaryngological, skin, hematological, surgical, and addiction disorders. Although fatigue, pain, and anxiety being common symptoms that impact the quality of life of MG patients, there was no consensus to include such data, resulting in the exclusion of all the proposed scales: Fatigue Severity Scale, PROMIS Fatigue Score, Hospital Anxiety and Depression Scale [HADS], Insomnia Severity Index [ISI], and Visual Analogue Pain Scale. The SF-36, despite its widespread use, was excluded due to concerns about its time-consuming nature and lack of specificity for MG.

7 items achieved ≥ 80% agreement for inclusion but did not reach ≥ 70% consensus for classification as either MD or OD. These items are considered of major interest but require further standardization. A complete list of excluded and unclassified items is provided in Table 3.

### Frequency of data collection

The experts agreed on collecting data on an annual basis to ensure consistent data collection and facilitate longitudinal monitoring. This approach aims to maintain high-quality and comprehensive data over time.

## Discussion

Myasthenia gravis (MG) is a rare and treatable autoimmune disorder. Over the past decade, the therapeutic landscape for MG has expanded significantly [32, 39, 40]. Advances in our understanding of the pathophysiology of MG have facilitated the development of new targeted therapeutics that exhibit more favorable side effect profiles [41]. This progress not only enhances treatment options for patients but also underscores the necessity for comprehensive registries that can effectively track the long-term outcomes and safety profiles of these emerging therapies. By integrating these advancements into our data collection efforts, we can better evaluate the impact of new treatments on patient health and optimize management strategies.

Moreover, the introduction of new therapies highlights the critical need to collect and share data safely among stakeholders [42, 43]. Such collaboration will deepen our understanding of the disease, improve patient outcomes, and assess the long-term effects of these innovative treatments [44]. Although several national MG registries have been launched across Europe, disparities persist between countries that cannot be fully captured by these national registries. A more collaborative, pan-European framework is needed to address these differences, especially in the context of unequal access to novel therapies driven by high costs and varying reimbursement criteria across Europe. Additionally, socio-economic, medico-economic, and epidemiological factors further contribute to the disparities in MG management, leading to health inequities across Europe.

The sharing of high-quality, aggregated, and harmonized data between national registries could greatly assist in ensuring more equitable management of MG patients across Europe. However, several challenges must be addressed to achieve this goal. Beyond resolving technical, ethical, and legal issues, the first step toward European collaboration is the harmonization of key data elements that must be collected and shared. This was the objective of the present study, which leveraged a multidisciplinary collaboration of experts and stakeholders to ensure that all relevant issues were addressed before defining the dataset. This also fostered an important dialogue on the necessity of data sharing.

To facilitate data collection, the study distinguished between MD and OD, prioritizing essential items while increasing the likelihood that the most important data are collected consistently. Based on input from patient associations, a specific session on the ICF was included, as patient representatives emphasized the need for transparency regarding data collection and use. Although the ERN had already established guidelines for rare disease registries in 2021 [45, 46] many MG registries had not fully adhered to these standards. Most of the MG registries were established prior to the publication of the ERN ICF [19, 47], and no harmonization has taken place thus far. A key point of debate was the inclusion of the item "data use for commercial purposes." Experts were concerned that patients might be reluctant to share their data with pharmaceutical companies, which could impact their willingness to participate in the registry. Ultimately, the panel agreed that the formulation of this section in the ICF should carefully outline the purposes and conditions of data sharing. Another critical discussion revolved around the removal of direct patient identifiers and the need to carefully assess and modify indirect identifiers to protect patient privacy and sensitive data.

In addition to demographic, diagnostic, clinical, and treatment-related data, consensus was reached on clinical outcome measures that would help evaluate the efficacy of therapeutic approaches. The MG-ADL score and the clinician-reported MGFA-D emerged as the mandatory severity measures to be collected, while other scores, such as the QMG score, MGC-score, and MGFA-CS, were classified as OD. The MG-ADL score is an essential tool for assessing the impact of treatment on a patient’s ability to perform daily activities and has been widely used as a primary outcome measure in clinical trials. A 2-point improvement in this score is generally considered clinically significant [48]. However, it has limitations, including its inability to capture the burden of treatment side effects, administration constraints, and several unassessed symptoms of MG [38]. Additionally, patients experiencing severe MG crises may be unable to complete the form. Fatigue, a core symptom of MG, is not directly measured by the MG-ADL.

Despite proposing various scores to measure fatigue (FSS, PROMIS Fatigue), none were retained in the final dataset, highlighting the need for a more specific and simple measure of fatigue for routine use in MG. Similarly, pain and anxiety, frequently reported by MG patients, were not included in the final dataset, despite advocacy from patient associations. While these symptoms can significantly impact quality of life, no validated measures were agreed upon. An indirect assessment of these symptoms may be obtained from the MG-QoL15r, which was included as OD in the final dataset.

Given the importance of comparing the efficacy and safety of different MG therapies, all data related to MG treatments (i.e. class, name and dosage) were included in the final dataset as OD. However, detailed information on non-MG-related pharmacological therapies and non-pharmacological treatments (e.g. physiotherapy, psychological support) were excluded. Although concurrent therapies are important for minimizing risks such as polypharmacy and for optimizing care, it was determined that these data should be captured through hospital records. The expert panel also agreed to include certain comorbidities as OD, particularly those most frequently associated with MG or impacting on treatment decisions. Several Delphi rounds were conducted to finalize the selection of comorbidities, resulting in the exclusion of psychiatric, gastrointestinal, and ophthalmological conditions due to concerns about the registry’s length.

Other demographic information such as city of residence, education, social factors, and clinician experience, were also excluded. While these elements could help identify healthcare disparities and influence diagnostic and treatment decisions, they were not prioritized. Ethnic origins were similarly excluded despite evidence suggesting ethnic variation in disease prevalence, treatment response, and genetic susceptibility to auto-immune disorders [49].

To achieve a balance between feasibility and data collection quality, certain compromises were necessary. Specifically, several items of interest were omitted to prioritize the collection of essential data deemed critical for the registry of the network of registries. This focus is vital for harmonizing data elements gathered from patients across various centers and registries, ultimately facilitating the establishment of a unified data quality system [50]. Despite these adjustments, the final dataset remains consistent with existing frameworks for data elements and adheres to the recommendations outlined in the EMA's guidelines on rare disease registries. This alignment is essential for effective registry management and the optimal use of collected data.

Several issues remain to be addressed, particularly regarding privacy and data protection legislation across different European countries. Technical, ethical, and legal challenges must also be overcome to enable data sharing at the European level. Future initiatives should build on this study’s work and further explore these issues to facilitate broader European and international collaboration.

## Conclusions

A consensus was achieved through a modified Delphi procedure involving a multidisciplinary panel of European experts on MG. This expert-driven approach marks a significant step toward data harmonization among European national MG registries. The resulting dataset will serve as a foundation for establishing a European collaboration on MG, addressing key clinical, research, and regulatory questions, and supporting decisions on treatment availability across European countries.

## Data Availability

Data available on request.

## Abbreviations

AChR: Acetylcholine receptor
AFM-Téléthon: French Muscular Dystrophy Association
AMES: Asociación Miastenia de España
EMA: European Medicines Agency
ENMC: European Neuro Muscular Centre
ERN: European Reference Network
ERN-NMD: European Reference Network for Neuromuscular Diseases
EURORDIS: Rare Diseases Europe
FSS: Fatigue Severity Scale
GDPR: General data protection regulation
HADS: Hospital anxiety and depression scale
ICF: Informed consent form
ISI: Insomnia Severity Index
LRP4: Lipoprotein-related protein 4
MD: Mandatory data
MG: Myasthenia gravis
MG-ADL: Myasthenia gravis activities of daily living
MGFA: Myasthenia gravis foundation of America
MGFA-CS: Myasthenia gravis foundation of America clinical status
MGFA-D: Myasthenia gravis foundation of America classification at diagnosis
MGC: Myasthenia gravis composite
MG-QoL15r: Myasthenia gravis quality of life 15-item revised
MuSK: Muscle-specific tyrosine kinase
NMJ: Neuromuscular junction
OD: Optional data
PROMIS Fatigue: Patient Reported Outcome Measurement Information System -Fatigue
QMG: Quantitative Myasthenia Gravis
SF-36: 36-Item Short Form Survey
